# Digital use of standardised assessment tools for children and adolescents: can available paper-based questionnaires be used free of charge in electronic format?

**DOI:** 10.1186/s12888-022-04023-w

**Published:** 2022-06-03

**Authors:** Marianne Cottin, Kathrin Blum, Jon Konjufca, Yamil Quevedo, Sylvia Kaaya, Alex Behn, Klaus Schmeck, Carla Sharp, Ronan Zimmermann

**Affiliations:** 1 Millennium Institute for Research in Depression and Personality (MIDAP), Santiago, Chile; 2 Department of Psychiatry East Campus, Faculty of Medicine, University of Chile, Santiago, Chile; 3 School of Psychology, Finis Terrae University, Santiago, Chile; 4 School of Psychology, Pontificia Universidad Católica de Chile, Santiago, Chile; 5 Child and Adolescent Psychiatric Research Department, Psychiatric University Hospitals of the University of Basel, Basel, Switzerland; 6 University of Prishtina, Pristina, Kosovo; 7 Division of Clinical Psychology and Psychotherapy, Faculty of Psychology, University of Basel, Basel, Switzerland; 8 Department of Psychiatry and Mental Health, School of Medicine, Muhimbili University of Health and Allied Science, Dar es Salaam, Tanzania; 9 Department of Psychology, University of Houston, Houston, TX USA

**Keywords:** Digitalization, Evidence-based assessment, Low-income contexts, Mental health, children, Adolescents

## Abstract

**Question:**

Most adolescents live in low- and middle-income countries (LMIC), and about 10% of them face mental problems. The mental health provision gap in low- and middle-income countries could be addressed by evidence-based practices, however costs are implementational barriers. Digitalization can improve the accessibility of these tools and constitutes a chance for LMIC to use them more easily at a low cost. We reviewed free and brief evidence-based mental health assessment tools available for digital use to assess psychopathology across different domains in youth.

**Methods:**

For the current study, instruments from a recent review on paper-based instruments were re-used. Additionally, a systematic search was conducted to add instruments for the personality disorder domain. We searched and classified the copyright and license terms available from the internet in terms of free usage and deliverability in a digital format. In the case that this information was insufficient, we contacted the authors.

**Results:**

In total, we evaluated 109 instruments. Of these instruments, 53 were free and digitally usable covering 11 mental health domains. However, retrieving information on copyright and license terms was very difficult.

**Conclusions:**

Free and digitally adaptable instruments are available, supporting the strategy of using instruments digitally to increase access. The instrument’s authors support this initiative, however, the lack of copyright information and the difficulties in contacting the authors and licence holders are barriers to using this strategy in LMIC. A comprehensive, online instrument repository for clinical practice would be an appropriate next step to make the instruments more accessible and reduce implementation barriers.

**Supplementary Information:**

The online version contains supplementary material available at 10.1186/s12888-022-04023-w.

## Background

The estimated pooled global point prevalence of mental disorders in youth is 13.4% [1, 2], and most adult neuropsychiatric disorders originate during childhood or adolescence [3]. Functional impairment in youth is likely to compromise life in adulthood [4, 5]. Thus, addressing mental health problems early in life is an opportunity and priority for the global mental health agenda [6, 7]. Ninety percent of youth around the world live in low-and-middle-income countries (LMIC) [8], where mental disorders have an exceptionally high prevalence [9]. Developing adequate mental health services is problematic due to a lack of government policy, inadequate funding, and insufficient trained clinicians [6]. Cost-efficient strategies to address this mental health care gap in lower resource contexts are urgently needed [10, 11]. In general, health professionals often make decisions about diagnosis and treatment under stressful circumstances and with incomplete information [12].

Evidence-based assessment (EBA) is an essential component of improving mental health care services. EBA is an approach to clinical evaluations “[...] that uses research and theory to guide the selection of constructs to be assessed for a specific assessment purpose, the methods and measures to be used in the assessment, and the manner in which the assessment process unfolds” [13]. EBA relies on the use of psychometrically sound instruments and clinical decision-making algorithms [14]. Importantly, EBA can act as a structuring principle by guiding initial case formulation, helping identify treatment targets, and selecting effective treatment strategies.

EBA can also support treatment monitoring which constitutes a critical feedback loop for health care workers [14]. The use of EBA is associated with improved treatment engagement and treatment response in youth [15, 16]. EBA can optimize care in non-specialized settings, especially in combination with task-shifting strategies to overcome the lack of trained personnel [10]. However, the cost and logistics involved in instrument selection, delivery, and interpretation present barriers to implementing EBA [17].

While paper-and-pencil formats provide an inexpensive solution, digital formats can be advantageous, structuring EBA and increasing efficiency through the automatization of scale calculation and retrieval of normative values. Automatically generated reports can provide guidance for the next steps in treatment. Integrating a digital EBA system with an electronic health record (EHR) can help cover clinical and administrative processes and streamline clinical care. We expect that digitalized EBA can support and improve the quality of mental health care without burdening healthcare workers.

Digital literacy and access to the internet have vastly expanded, particularly in LMICs [18]. Moreover, children and adolescents are likely to be more familiar with digital formats on computers and mobile devices and find them easier to use and more appealing than paper-and-pencil formats [19]. This trend will likely become even more accentuated in the future. Digitalization is a foundation for data-driven improvements of health care services, for example, through data-driven research and improved quality management. However, the use and benefit of e-tools is far from matching its potential [20].

The Mental Health Information Reporting Assistant (MHIRA) project is working on developing and implementing digital EBA in LMICs [21]. Our goal is to provide an open-source software platform to support digital EBA, making EBA more accessible and affordable in LMICs. For digital development, many institutions involved in global health, including USAID and World Health Organisation, endorse the nine design principles for digital development: “design with the user”; “understand the existing ecosystem”; “design for scale”; “build for sustainability”; “be data driven”; “use open standards”, “open data”, “open source and open innovation”; “reuse and improve”; “address privacy and security”; and “be collaborative” [22]. MHIRA follows these principles. In line with the principle “reuse and improve”, the MHIRA research group hypothesizes that many psychometric instruments available in paper-pencil format can be freely adapted and used in an electronic format.

### Free use in digital formats

Digital versions of psychometric instruments of child and adolescent psychopathology (e.g., Achenbach scales or the BASC) are available. However, these solutions require a license fee, and while the fee might be justified compared to the benefits for the health care services, costs undoubtedly present a barrier in LMICs. Providing free access to EBA instrumentation is crucial in such settings.

In this context, Becker-Haimes et al. [17] found 95 free and brief instruments (in paper-and-pencil format) to assess overall mental health, depression, eating disorders, anxiety, substance use, disruptive behaviour, suicidality, bipolar disorder, and psychosis in youth. However, the list of instruments is not necessarily valid for the digital implementation of the instruments. “Free” instruments might have license terms that do not allow for free digital usage; that is, only a paper-and-pencil version is free for use.

The aim of the current review was to identify brief and free instruments that can be used to assess common mental health as well as personality disorders in children and adolescents in a digital format.

We focused on brief instruments because they are less time-consuming for clinicians, and less burdening for patients. We hypothesized that sufficient authors and copyright holders would allow for free digital usage of instruments to cover the assessment of a wide spectrum of mental health disorders in children and adolescents. Additionally, we investigated the availability and quality of information to determine whether healthcare workers could use an instrument for free in a digitally adapted format.

The increasing use of mobile devices, the proliferation of digital technologies, and the development of e-health services in response to rising demand have created great expectations and opportunities for the potential of health care delivered using digital tools. This review is a timely first step in increasing use of EBA to help address current global mental health needs.

## Methods

### *Updating the list compiled by Becker-Haimes* et al. [17]

Reusing and building on currently available resources is a principle to apply digital technologies to development programs. Hence, we reused results from a recently published systematic review by Becker-Haimes et al. [17] with the new purpose of collecting information about free usage in digital format. In their synthesis, Becker-Haimes et al. [17] included 95 brief and free instruments for the assessment of mental health in children and adolescents distributed in 10 domains: Overall mental health *n* = 12; Anxiety *n* = 11; Depression *n* = 13; Disruptive behaviour *n =* 12; Trauma *n* = 7; Eating disorders *n =* 12; Suicidality *n* = 6; Bipolar disorders *n =* 6; Psychosis *n* = 3; Substance use *n* = 13.

### Addition of measures for personality disorders

In addition to these, we included the domain of personality disorders. The diagnostic system for personality disorders is shifting towards a dimensional approach. The current review only considers the newer dimensional approach reflected in the upcoming ICD-11, mandatory starting in 2022, and the Alternative Model for Personality Disorders (AMPD) of the DSM-5 [23, 24].

First, we adopted the search criteria used by Becker-Haimes et al. [17] to identify standardized instruments for personality assessment. We searched the PubMed and PsycINFO databases from 1986 to September 2020 using the following search terms: ("Personality Disorder" OR "Borderline personality disorder") AND ("instrument" OR "survey" OR "questionnaire" OR "measure" OR "assessment") AND ("psychometric" OR "measure development" OR "measure validation“). We filtered by age criteria to include adolescents (13-18 years) and young adults (19-24 years). We considered all empirical studies and reviews of personality measures for children and adolescents.

We then screened titles and abstracts. We classified articles as “relevant” if they reported instruments that were brief, i.e., 50 items or fewer, including sub-scale items to be consistent with the review by Becker-Haimes et al. [17]. We reviewed articles that described a self-or-third party-report measure for evaluating personality disorders based on the ICD-11 or DSM-5 AMPD (i.e., papers that referenced scales assessing personality functioning, maladaptive personality traits, and borderline personality disorder). To comply with the dimensional approach using a general personality disorder category, we excluded instruments for specific personality disorders according to the categorical approach (e.g., narcissistic personality disorder). However, we retained instruments for borderline personality disorder because, according to the ICD-11 criteria, it still constitutes a qualifier based on trait configuration, a diagnostic procedure also valid for DSM-5 AMPD. We excluded instruments that referenced measures for assessing components of personality pathology but not for diagnosis (e.g., emotion regulation, impulsivity). When in doubt, we decided on the relevance of articles in consensus meetings of the authors MC and YQ. Finally, we excluded articles that were not in English. We added to the resulting list measures regularly used by authors and colleagues with expertise on personality disorders. The reason for this decision was that we wanted to acknowledge the clinical relevance of these measures, which is essential in the EBA approach. We did not register our protocol.

### Classifying instruments as “free to use and in digital format”

To examine whether instruments were free to use in digital format, we made basic considerations about copyright and license terms. To be considered an appropriate instrument for our purposes, the use of an instrument needed to not be contingent on payment. In addition, the adaptation to a digital format must be allowed, as this legally constitutes the creation of a derivative, which the authors had to explicitly authorize. Creative Commons is a global non-profit organization providing internationally applicable copyright license terms [25]. Based on these terms, we also distinguished between instruments that were free to use for commercial versus non-commercial purposes. We considered only commercially usable instruments to include in our final list. We made this decision considering that health care services will be the final users of the list of instruments in mental health settings where patients might have to pay for these services. Instruments in the public domain (i.e., no exclusive intellectual property rights apply) to meet our “Public Domain” criteria were included [26].

### Copyright search procedure

First, we researched copyright and license terms from instrument-related publications or copies of the instruments we could find. If not available from there, we searched for copyright information on Google combining the name of each instrument with the search terms “copyright” and “license”. When we could not make a clear assessment based on the retrieved information or when copyright information was unavailable (see additional materials), we sent two standardized emails to the authors within 2 months. In these emails, we asked permission from the authors to use their instruments for our purposes (i.e., free clinical use and adaptation to digital format). We found contact information using one of the following methods a) from the instrument-related publication, b) from a Google search using the author’s name as a search term, or c) from the ResearchGate platform [27]. We excluded instruments from our list in case we could not contact the authors. A few responses were unclear (e.g., authors explicitly allowed clinical use but not commercial use), in which case we contacted the authors again for further clarification. We refrained from any further clarification attempts after this for feasibility reasons. In the next step, we analyzed all copyright information from the different sources regarding the two criteria of our review (i.e., free clinical use and adaptation to digital format).

At this point, we sorted out cases that remained unclear and excluded instruments whenever we did not find viable options for using the instruments in a digital format or when the authors could only grant permission under a standardized procedure for the eventual end-user (i.e., individual clinicians or clinics). Finally, we excluded instruments that were only allowed for use by specific end-user subgroups (e.g., only individual practitioners but not clinics, or only specific countries).

## Results

In this review, we started from two sources to compile a final list of free and brief instruments for clinical use in a digital format, one based on a reused list by Becker-Haimes [17] and the other based on our systematic search of the literature on instruments for the assessment of personality disorders. Regarding our first source, we added one instrument for assessing childhood trauma (‘Child Trauma Screen’ Questionnaire) which met our inclusion criteria and was pointed out to us when contacting the authors of another measure. The revised list comprised 96 instruments for the assessment of different psychopathology domains. Our systematic search of the literature on personality disorder instruments resulted in 69 articles that presented 59 additional instruments (refer to Supplementary File 2 for the PRISMA Flow Diagram). Fifty instruments were removed because they did not meet inclusion criteria (e.g., containing less than 50 items or were not tested in children or adolescents, etc.). This resulted in a list of 9 instruments to which 3 instruments were added by experts collaborating in the project. A final list of 13 instruments for the assessment of personality disorders were identified. Two instruments assessed Criterion A (i.e., personality functioning), two assessed Criterion B (i.e., maladaptive personality traits), and eight evaluated borderline personality disorder.

After merging the two sources, we evaluated 109 instruments regarding copyright licensing conditions (refer to the list of instruments in Supplementary File 1). This list did not count versions with different numbers of items. To finally decide whether instruments were allowed to be used for free in clinical settings and adapted into a digital format, we assessed accessible copyright information from the internet or the copyrights holders’ statements in their email responses.

Our analysis of the copyright license information found on the internet (without considering the email responses of the copyright holders) revealed that 30 instruments were usable in clinical routine without costs. However, only 14 instruments were usable in clinical routine and adaptable to a digital version. For 59 instruments, the information retrieved from the internet was not sufficient regarding free usage. For 86 instruments, it was not clear whether the license holder allowed the adaptation of the instrument to a digital format. For details on single instruments, please refer to Supplementary File 1 which summarizes the information retrieved on the internet regarding the usage terms for each scale.

For 88 instruments (81%), the information found on the internet regarding free digital usage was not conclusive. Consequently, we contacted the copyright holders of these instruments (see Supplementary File 3). Out of the 88 contacted copyright holders, 39 allowed using their instrument for the described use case of distributing the instrument in a free open-source platform. Only four copyright holders did not allow free usage in digital format. There was no definite answer in five cases because the current review is a hypothetical case and authors only gave permission case by case based on a formal application process. In 33 cases (33%) we were unable to reach the copyright holders due to lack of contact information, or we did not receive a response to our inquiry. In the next section, these cases are referred to as “no use possible” because the instruments should not be used without the permission of the copyright holder.

**Fig. 1:**
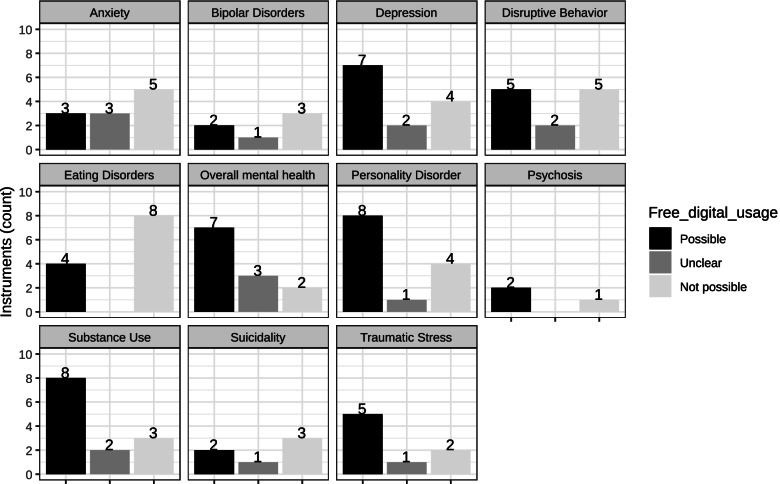
Main result - Count of free digitally usable instruments. Figure 1 shows how many instruments were adaptable to a digital format and free to use in the investigated psychopathology domains

Our final list of instruments that can be freely used in clinical settings and adapted to a digital format comprised 53 instruments for the assessment of 11 domains (please see Fig. 1): overall mental health *n* = 7; anxiety *n* = 3; depression *n =* 7; disruptive behaviour *n* = 5; traumatic stress *n =* 5; eating disorders *n =* 4; suicidality *n* = 2; bipolar disorders *n =* 2; psychosis *n =* 2; substance use *n* = 8; and personality disorders *n* = 8. Supplementary File 1 shows the instruments for each included domain. At least two instruments were free and adaptable to digital format for each domain. For some domains, up to 8 free and adaptable instruments were available.

## Discussion

A minimum of two and a maximum of eight instruments were freely usable in digital format for each investigated psychopathology domain. We received many positive and encouraging email responses from copyright holders indicating that they are very supportive of using their instruments in regular clinical work or research free of charge. The only consistent request made by authors was a correct citation of the instrument authorship.

However, accessing license and copyright information was a major hurdle: it is complex and time-consuming for clinicians to determine if they can freely use a given instrument in a digital format. For the criterion “free usage” alone, the information was not conclusive for 50% of the instruments. For the combined features “free usage” and “permission to use software adapted format” information was insufficient in 81% of the instruments. Even after attempting to contact the authors, the information was still insufficient for 33% of the instruments due to unavailable contact information or no response.

Copyright information would be most readily available if the authors provided this information on the instruments or in a corresponding scientific article. Instruments with published copyright statements can be used according to the terms indicated in the copyright statement without contacting the copyright holder. However, by default, the status of instruments without a published copyright statement is that all rights are reserved by the copyright holder. Therefore, health care workers interested in these instruments will need to contact the copyright holders to get permission. In the list of instruments in Supplementary File 1, instruments with a published copyright statement can be found consulting the columns “Free (copyright licence info available on the internet)” and “Software adaptation allowed (copyright licence info available on the internet)”. In contrast, for those instruments for which we received approval via email, the copyright holders are open to grant free access and allow digital usage, however, we recommend that written permission be obtained from the copyright holder.

In summary, it required considerable effort to find free instruments with reliable information on their usage terms. We argue that this is a barrier to the use of digital and paper-based EBA in LMICs, especially as mental health workers in LMIC may be less well connected through research networks to stay up to date on current EBA. An online repository for clinical practice instruments would be a convenient solution to this problem. It could centralize all required information by, for example, offering more straightforward ways to contact the authors or publishers of instruments or containing structured information about usability terms. Additionally, a repository could address the problem of source legitimacy. The uploaded instruments could require a Common Creative license, thus standardizing the copyright issue, and adding instruments would require a confirmation that the user has permission from the copyright holder. Moreover, individuals may report instruments that are incorrectly uploaded.

Another issue is the quality of instruments, their clinical utility, and contextual information about their optimal use. The psychometric properties of all instruments in Supplementary File 1 are discussed in either [17, 28–30], with the exception of the personality disorder assessment instruments, most of which are found in either [31, 32]. However, in our view, reviews are not the most convenient resource on instrument quality information because they cannot be updated. Researchers must conduct a new review using their own search terms and criteria for instrument selection, which is inefficient. An online repository providing psychometric properties and links to the original articles could help clinicians select appropriate brief and free instruments in digital formats that meet their needs and are appropriate for the relevant population. In such a repository, clinicians could also curate instruments and leave comments (as is already familiar from well-known online platforms) on the quality and clinical utility of the instruments as additional information. Users could also report on their experience with the instruments and give advice on how to best use the instrument. Finally, a search engine would enable filtering instruments by age range, informant perspective, topic, disorder, and other relevant criteria.

A significant limitation of the current study and other reviews is restricting searches to English versions of the instruments. Often, authors do not provide information on cultural adaptations and their respective psychometric properties (e.g., Becker-Haimes et al. [17]). This limitation makes it difficult to find available instruments for non-English speaking contexts. Information on available cultural adaptations is complicated to provide. Again, a repository could contain this information, and a filter could point users to the available culturally adapted instruments. This platform would facilitate an instrument exchange between clinicians and researchers who could share their work. A relevant question is who should offer such a repository to guarantee sustainability and legitimacy to the service.

An open-source digital format would likely be a good solution for digital versions. Open-source software projects could provide helpful functionalities, such as validating the entered data and skipping non-relevant questions based on previous responses, which could be considered a standard. Individuals could create questionnaires using a spreadsheet or different questionnaire builders. Even though such open-source tools are used in research, they are less so in mental health clinical applications.

The use of instruments in a digital format presents several challenges for mental health services. Barriers include high costs, the need for adequate training, and ensuring data security and privacy in data storage and transfer [33, 34]. Although these technical and practical limitations exist, digital assessments offer the advantages of speed in administration, automated scoring, quick delivery of results, and the possibility of reaching remote locations more easily than face-to-face assessments. Digital assessments are also more efficient when scaling, because increasing volume does not significantly increase costs [30].

Implementing EBA practices across clinical settings and delivering assessment instruments in digital formats requires specialized software. Our motivation for writing this review is our participation in the MHIRA project [21]. The project revolves around a digital platform that facilitates data-driven and evidence-based clinical practice to support mental health care workers, including and especially in LMICs. The intention behind MHIRA is also to facilitate structured data collection for research. By following the digital principle of ‘reuse and improve’, MHIRA can make existing instruments available digitally. The software is open-source (https://github.com/mhira-project/) and accessible in-low resource contexts with initial implementation sites in Latin America, Eastern Europe, and Sub-Saharan Africa. This review addresses the availability of free EBA for digital usage for MHIRA or similar software.

When transitioning from a paper-and-pencil to a digital format, the validity of previously established psychometric properties must be carefully considered [31]. However, paper and digital versions can be considered equivalent under certain conditions [32]. Collecting normative data with digital versions and comparison to paper versions are required.

## Conclusion

Instruments that can be delivered free of charge in a digital format do exist and are available for clinical and scientific use in several domains of psychopathology. However, access to copyright information remains challenging. This may hinder the use of available instruments and delay the implementation of urgently needed evidence-based assessment procedures. Instrument developers should strive to provide clear copyright information including details on adaptations to the digital format. Open-access software for providing and reporting free and digital instruments is needed.

## Supplementary Information

**Additional file 1.**

**Additional file 2.**

**Additional file 3.**

## Data Availability

All data generated and analysed during this study are included in this published article and its supplementary information files (Supplementary Files 1, 2 and 3).

## Abbreviations

AMPD: Alternative Model for Personality Disorders
EBA: Evidence – based assessment
EHR: Electronic health record
LMIC: Low- and middle-income countries
MHIRA: Mental Health Information Assistant

## References

[1] Fusar-Poli P, Hijazi Z, Stahl D, Steyerberg EW (2018). The science of prognosis in psychiatry: a review. JAMA Psychiatry.

[2] Van Scotter JR, Roglio KDD (2018). Ceo bright and dark personality: effects on ethical misconduct. J Bus Ethics.

[3] Lund C (2018). Improving quality of mental health care in low-resource settings: lessons from PRIME. World Psychiatry.

[4] Bloom DE, Cafiero E, Jané-Llopis E, Abrahams-Gessel S, Bloom LR, Fathima S, et al. The global economic burden of noncommunicable diseases [Internet]. PGDA Working Papers. Geneva: Program on the Global Demography of Aging. 2012; Available from: https://ideas.repec.org/p/gdm/wpaper/8712.html. [cited 2020 Jun 22]. (PGDA Working Papers). Report No.: 8712.

[5] Sawyer SM, Afifi RA, Bearinger LH, Blakemore S-J, Dick B, Ezeh AC (2012). Adolescence: a foundation for future health. Lancet.

[6] Kieling C, Baker-Henningham H, Belfer M, Conti G, Ertem I, Omigbodun O (2011). Child and adolescent mental health worldwide: evidence for action. Lancet..

[7] Dahl RE, Allen NB, Wilbrecht L, Suleiman AB (2018). Importance of investing in adolescence from a developmental science perspective. Nature..

[8] United Nations (2017). World population prospects: the 2017 revision, key findings and advance tables [internet].

[9] Pedersen G, Arnevik EA, Hummelen B, Walderhaug E, Wilberg T (2019). Psychometric properties of the severity indices of personality problems (SIPP) in two samples: a Norwegian community sample and clinical samples of patients with and without personality disorders. Eur J Psychol Assess.

[10] Dorsey S, Gray CL, Wasonga AI, Amanya C, Weiner BJ, Belden CM (2020). Advancing successful implementation of task-shifted mental health care in low-resource settings (BASIC): protocol for a stepped wedge cluster randomized trial. BMC Psychiatry.

[11] Thornicroft G, Alem A, Dos Santos RA, Barley E, Drake RE, Gregorio G (2010). WPA guidance on steps, obstacles and mistakes to avoid in the implementation of community mental health care. World Psychiatry.

[12] Galanter CA, Patel VL (2005). Medical decision making: a selective review for child psychiatrists and psychologists. J Child Psychol Psychiatry.

[13] Hunsley J, Mash EJ (2007). Evidence-based assessment. Annu Rev Clin Psychol.

[14] Youngstrom EA, Van MA, Frazier TW, Hunsley J, Prinstein MJ, Ong M-L (2017). Evidence-based assessment as an integrative model for applying psychological science to guide the voyage of treatment. Clin Psychol Sci Pract.

[15] Pogge DL, Wayland-Smith D, Zaccario M, Borgaro S, Stokes J, Harvey PD (2001). Diagnosis of manic episodes in adolescent inpatients: structured diagnostic procedures compared to clinical chart diagnoses. Psychiatry Res.

[16] Klein JB, Lavigne JV, Seshadri R (2010). Clinician-assigned and parent-report questionnaire-derived child psychiatric diagnoses: correlates and consequences of disagreement. Am J Orthop.

[17] Becker-Haimes EM, Tabachnick AR, Last BS, Stewart RE, Hasan-Granier A, Beidas RS (2020). Evidence base update for brief, free, and accessible youth mental health measures. J Clin Child Adolesc Psychol.

[18] Makri A (2019). Bridging the digital divide in health care. Lancet Digit Health.

[19] Varni JW, Magnus B, Stucky BD, Liu Y, Quinn H, Thissen D (2014). Psychometric properties of the PROMIS ® pediatric scales: precision, stability, and comparison of different scoring and administration options. Qual Life Res.

[20] Roberts LW, Chan S, Torous J (2018). New tests, new tools: mobile and connected technologies in advancing psychiatric diagnosis. npj Digit Med.

[21] Mental Health Information Reporting Assistant. https://mhira-project.org. Accessed 26 January 2022.

[22] Principles for Digital Development. https://digitalprinciples.org. Accessed 26 January 2022.

[23] Sharp C, Fonagy P (2015). Practitioner review: borderline personality disorder in adolescence - recent conceptualization, intervention, and implications for clinical practice. J Child Psychol Psychiatry.

[24] Sharp C, Kerr S, Chanen A, Skodol AE, Oldham JM (2021). Early identification and prevention of personality pathology: An AMPD-informed model of clinical staging. The American Psychiatric Association publishing textbook of personality disorders.

[25] Creative Commons. https://creativecommons.org/. Accessed 26 January 2022.

[26] Creative Commons: Public Domain. https://creativecommons.org/share-your-work/public-domain/. Accessed 26 January 2022.

[27] Research Gate. https://www.researchgate.net/. Accessed 26 January 2022.

[28] Lustgarten SD, Garrison YL, Sinnard MT, Flynn AWP (2020). Digital privacy in mental healthcare: current issues and recommendations for technology use. Curr Opin Psychol.

[29] Timmis S, Broadfoot P, Sutherland R, Oldfield A (2016). Rethinking assessment in a digital age: opportunities, challenges and risks. Br Educ Res J.

[30] Naglieri JA, Drasgow F, Schmit M, Handler L, Prifitera A, Margolis A (2004). Psychological testing on the internet: new problems. Old Issues Am Psychol.

[31] Coons SJ, Gwaltney CJ, Hays RD, Lundy JJ, Sloan JA, Revicki DA (2009). Recommendations on evidence needed to support measurement equivalence between electronic and paper-based patient-reported outcome (PRO) measures: ISPOR ePRO good research practices task force report. Value Health.

[32] van Ballegooijen W, Riper H, Cuijpers P, van Oppen P, Smit JH (2016). Validation of online psychometric instruments for common mental health disorders: a systematic review. BMC Psychiatry.

[33] Birkhölzer M, Schmeck K, Goth K (2021). Assessment of criterion a. Curr Opin Psychol.

[34] Zimmermann J, Kerber A, Rek K, Hopwood CJ, Krueger RF (2019). A brief but comprehensive review of research on the alternative DSM-5 model for personality disorders. Curr Psychiatry Rep.

