# Supplementary material for: Digital use of standardised assessment tools for children and adolescents: can available paper-based questionnaires be used free of charge in electronic format?
Source: BMC Psychiatry. 2022 Jun 3;22:379. doi: 10.1186/s12888-022-04023-w (PMC9166519; doi:10.1186/s12888-022-04023-w)
Supplement: Supplementary file 1 — Additional file 1. [file 12888_2022_4023_MOESM1_ESM.pdf]

Domain	Personality disorder sub-domain	Abbreviation	Measure Name	Free (copyright license info available on the internet)	Software adaptation allowed (copyright license info available on the internet)	Copyright holder permission to use as free online adaptation (resulting from e-mail answer)	Free usage in digital format (final descision)	Number of items	Informant	Age range	Clinic al Uses
Anxiety		CALIS/CALIS-PV	Child Anxiety Life Interference Scale-Parent/Child, form and preschool Version	Clinical use allowed	No	Contacting author not required	Not possible	19, 10, 18	Y, P	3 - 17	D, O
Anxiety		HAM-A	Hamilton Anxiety Rating Scale-A	Clinical use allowed	Yes	Contacting author not required	Possible	14	C	11 - 18	D, O
Anxiety		PAS	Preschool Anxiety Scale-Revised	No sufficient information	Yes	Unclear	Unclear	28	P	3 - 5	D
Anxiety		PROMIS Anxiety	Patient-Reported Outcomes Measurement Information System	Special conditions	Yes	Unclear	Unclear	15	Y, P	8 - 17	S, O
Anxiety		PSWQ-C	Penn State Worry Questionnaire-Child version	No sufficient information	No	Contacting author not required	Not possible	14	Y	7 - 17	D
Anxiety		RCADS; RCADS-P	Revised Child Anxiety and Depression Scale	No sufficient information	No	Contacting author not required	Not possible	25, 47	Y, P	9 - 17	D, O
Anxiety		SAS-TR	School Anxiety Scale-Teacher Report	Clinical use allowed	No	Contacting author not required	Not possible	16	T	5 - 12	D
Anxiety		SCARED	Screen for Child Anxiety-Related Emotional Disorders	No sufficient information	No sufficient information	Permission granted	Possible	41	Y, P	7 - 17	S, D, O
Anxiety		SCAS	Spence Children's Anxiety Scale	Special conditions	No	Contacting author not required	Not possible	38	Y, P	7 - 19	D, O
Anxiety		SOCS	Short OCD Screener	No sufficient information	No sufficient information	Permission granted	Possible	7	Y	9 - 19	S
Anxiety		SWQ	Social Worries Questionnaire	No sufficient information	No sufficient information	Unclear	Unclear	10	Y, P, T	8 - 17	S
Bipolar Disorders		CMRS-P	Child Mania Rating Scale-Parent Version	Non-commercial use	Yes	Permission granted	Possible	21	P	9 - 15	S, D, O
Bipolar Disorders		P-MDQ	Mood Disorder Questionnaire-Parent Version	No sufficient information	No sufficient information	Permission granted	Possible	13	P	12 - 17	S, D
Bipolar Disorders		PGBI-10M	10-item Mania General Behavior Inventory	No sufficient information	No sufficient information	Failed contacting	Not possible	10	P	5 - 17	S
Bipolar Disorders		WERCAP	Washington Early Recognition Center Affectivity and Psychosi Screen	No sufficient information	No sufficient information	Failed contacting	Not possible	16	Y	15 - 24	S, D

Table A.1. List of all instruments with their classification regarding free usage and adaptability to digital format.
This table presents the final list of instruments built as an update on a recent review by Becker-Haimes et al.[17], who reported on free, brief, and accessible instruments for assessing mental health in adolescents. Y: Youth; P: Parent: C: Clinician; T: Teacher; D: Diagnosis; O: Outcome monitoring; S: Screening.

Table A.1. continued

Domain	Personality disorder sub-domain	Abbreviation	Measure Name	Free (copyright license info available on the internet)	Software adaptation allowed (copyright license info available on the internet)	Copyright holder permission to use as free online adaptation (resulting from e-mail answer)	Free usage in digital format (final descision)	Number of items	Informant	Age range	Clinical Uses
Bipolar Disorders		YMRS	Young Mania Rating Scale	No sufficient information	No sufficient information	Unclear	Unclear	11	Y, P, C	5 - 17	S, D, O
Bipolar Disorders		GBI	7 Up 7 Down Inventory (Brief General Behavior Inventory)	No sufficient information	No sufficient information	Failed contacting	Not possible	14	P	5 - 18	S, D
Depression		CES-DC	Center for Epidemiological Studies-Depression Scale for children	No sufficient information	No sufficient information	Failed contacting	Not possible	20	Y	6 - 18	S
Depression		DASS-21	Depression Anxiety and Stress Scale	Clinical use allowed	Yes	Contacting author not required	Possible	21	Y	12 - 12	D
Depression		DSRS	Depression Self-Rating Scale	No sufficient information	No sufficient information	Failed contacting	Not possible	18	Y	8 - 14	S, D
Depression		HADS	Hospital Anxiety and Depression Scale	Not free	No sufficient information	Permission not granted	Not possible	14	Y	12 - 12	S, D, O
Depression		HSC	Hopelessness Scale for Children	No sufficient information	No sufficient information	Permission granted	Possible	17	Y, C	6 - 13	D, O
Depression		KADS	Kutcher Adolescent Depression Scale	No sufficient information	No sufficient information	Permission granted	Possible	6, 11	Y	12 - 17	S, D, O
Depression		MFQ	Mood and Feelings Questionnaire	Clinical use allowed	No sufficient information	Failed contacting	Unclear	13, 33	Y, P	6 - 17	S, D, O
Depression		PANAS-C	Positive and Negative Affect Scale for Children	Non-commercial use	No sufficient information	Permission granted	Possible	27	Y	9 - 17	D
Depression		PFC	Preschool Feelings Checklist	No sufficient information	No sufficient information	Permission granted	Possible	16	P	3 - 6	S, O
Depression		PHQ-9	Patient Health Questionnaire-9	Clinical use allowed	Yes	Contacting author not required	Possible	9	Y	13 - 13	S, D, O
Depression		PROMIS	PROMIS® negative affect measures (Depression, Anxiety, and Anger)	Special conditions	No sufficient information	Unclear	Unclear	8	Y, P	5 - 17	S, 0
Depression		RCADS; RCADS-P	Revised Child Anxiety and Depression Scale	Non-commercial use	No	Contacting author not required	Not possible	25, 47	Y, P	9 - 18	D, O
Depression		SRQ-20	Mental Health Problems Self Report Questionnaire	Clinical use allowed	Yes	Contacting author not required	Possible	20	Y	11 - 18	S

Table A.1. List of all instruments with their classification regarding free usage and adaptability to digital format.
This table presents the final list of instruments built as an update on a recent review by Becker-Haimes et al.[17], who reported on free, brief, and accessible instruments for assessing mental health in adolescents. Y: Youth; P: Parent: C: Clinician; T: Teacher; D: Diagnosis; O: Outcome monitoring; S: Screening.

Table A.1. continued

Domain	Personality disorder sub-domain	Abbreviation	Measure Name	Free (copyright license info available on the internet)	Software adaptation allowed (copyright license info available on the internet)	Copyright holder permission to use as free online adaptation (resulting from e-mail answer)	Free usage in digital format (final descision)	Number of items	Informant	Age range			Clinical Uses
Disruptive Behavior		BPI-S	Behavior Problems Inventory-Short Form	Special conditions	No sufficient information	Permission granted	Possible	30	P	2	-	2	D
Disruptive Behavior		C-SHARP	Children's Scale of Hostility and Aggression: Reactive/Proactive	Clinical use allowed	No sufficient information	Permission granted	Possible	48	P	3	-	21	S, D
Disruptive Behavior		CDRS (CDRS-P or CDRS-T)	Conduct Disorder Rating Scale for parents, Conduct Disorder Rating Scale for teachers	Non-commercial use	Yes	Permission granted	Possible	12, 15	P, T	5	-	12	S
Disruptive Behavior		DAS	Delinquent Activities Scale	Non-commercial use	Yes	Unclear	Unclear	37	Y	11	-	18	S, D, O
Disruptive Behavior		DBDRS	Disruptive Behavior Disorders Rating Scale	No sufficient information	No sufficient information	Failed contacting	Not possible	45	P, T	3	-	13	S, D, O
Disruptive Behavior		ICU	Inventory of Callous and Unemotional Traits	Special conditions	No sufficient information	Failed contacting	Not possible	12, 24	Y, P, T	3	-	18	D
Disruptive Behavior		MOAS	Modified Overt Aggression Scale	No sufficient information	No sufficient information	Unclear	Unclear	16	P, T	5	-	18	O
Disruptive Behavior		OMS	Outburst Monitoring Scale	Special conditions	No sufficient information	Failed contacting	Not possible	20	P	12	-	17	D, O
Disruptive Behavior		SNAP-IV	Swanson, Nolan, and Pelham Rating Scale	No sufficient information	No sufficient information	Permission granted	Possible	18, 26	P, T	5	-	17	S, D
Disruptive Behavior		SWAN	Strengths and Weaknesses of ADHD Symptoms and Normal-behavior scale	No sufficient information	No sufficient information	Permission granted	Possible	18	P, T	5	-	18	D, O
Disruptive Behavior		VADTRS	Vanderbilt ADHD Diagnostic Teacher Rating Scale	No sufficient information	No sufficient information	Failed contacting	Not possible	43	T	4	-	12	S
Disruptive Behavior		IOWA-CRS	IOWA Conners Rating Scale	No sufficient information	No sufficient information	Failed contacting	Not possible	10	P, T	7	-	18	S, D
Eating Disorders		BCQ	Body Checking Questionnaire	No sufficient information	No sufficient information	Permission granted	Possible	23	Y	15	-	15	D
Eating Disorders		BITE	Bulimic Investigatory Test, Edinburgh	Special conditions	No sufficient information	Failed contacting	Not possible	33	Y	12	-	12	S, D, O

Table A.1. List of all instruments with their classification regarding free usage and adaptability to digital format.
This table presents the final list of instruments built as an update on a recent review by Becker-Haimes et al.[17], who reported on free, brief, and accessible instruments for assessing mental health in adolescents. Y: Youth; P: Parent: C: Clinician; T: Teacher; D: Diagnosis; O: Outcome monitoring; S: Screening.

Table A.1. continued

Domain	Personality disorder sub-domain	Abbreviation	Measure Name	Free (copyright license info available on the internet)	Software adaptation allowed (copyright license info available on the internet)	Copyright holder permission to use as free online adaptation (resulting from e-mail answer)	Free usage in digital format (final descision)	Number of items	Informant	Age range			Clinical Uses
Eating Disorders		BSQ	Body Shape Questionnaire	No sufficient information	No sufficient information	Permission granted	Possible	8, 16, 34	Y	14	-	14	S, D, O
Eating Disorders		ChEAT	Children's Eating Attitudes Test	No sufficient information	No sufficient information	Failed contacting	Not possible	26	Y	8	-	13	S, D
Eating Disorders		CIA	Clinical Impairment Assessment	No sufficient information	No sufficient information	Failed contacting	Not possible	16	Y	11	-	18	S, D
Eating Disorders		EAT	Eating Attitudes Test	No sufficient information	No sufficient information	Permission granted	Possible	26, 40	Y	13	-	13	S, D
Eating Disorders		EDDS	Eating Disorder Diagnostic Scale	No sufficient information	No sufficient information	Failed contacting	Not possible	22	Y	13	-	13	D
Eating Disorders		EDY-Q	Eating Disturbances in Youth Questionnaire	No sufficient information	No sufficient information	Failed contacting	Not possible	14	Y	8	-	13	D
Eating Disorders		IBSS-R	Ideal Body Stereotype Scale-Revised	No sufficient information	No sufficient information	Failed contacting	Not possible	6	Y	11	-	18	D, O
Eating Disorders		PASTAS	Physical Appearance State and Trait Anxiety Scale	No sufficient information	No sufficient information	Failed contacting	Not possible	17	Y	13	-	13	D
Eating Disorders		SATAQ-4	Sociocultural Attitudes Towards Appearance Questionnaire-4	Non-commercial use	No sufficient information	Failed contacting	Not possible	30	Y	11	-	18	D
Eating Disorders		YFAS-C	Dimensional Yale Food Addiction Scale for Children 2.0	Non-commercial use	Yes	Permission granted	Possible	25	Y	13	-	13	D
Overall mental health		BAC-A; BAC-C	Brief Assessment Checklist for Children; Brief Assessment Checklist for Adolescents	Clinical use allowed	No sufficient information	Failed contacting	Unclear	20	P	4	-	17	S, 0
Overall mental health		BFS	Behavior and Feelings Survey	Clinical use allowed	Yes	Contacting author not required	Possible	12	Y, P	7	-	15	O
Overall mental health		ECSA	Early Childhood Screening Assessment	Clinical use allowed	No sufficient information	Failed contacting	Unclear	22	P	1,5	-	5	S
Overall mental health		K6	Kessler Psychological Distress Scale	Clinical use allowed	No sufficient information	Permission granted	Possible	6	Y	11	-	18	S

Table A.1. List of all instruments with their classification regarding free usage and adaptability to digital format.
This table presents the final list of instruments built as an update on a recent review by Becker-Haimes et al.[17], who reported on free, brief, and accessible instruments for assessing mental health in adolescents. Y: Youth; P: Parent: C: Clinician; T: Teacher; D: Diagnosis; O: Outcome monitoring; S: Screening.

Table A.1. continued

Domain	Personality disorder sub-domain	Abbreviation	Measure Name	Free (copyright license info available on the internet)	Software adaptation allowed (copyright license info available on the internet)	Copyright holder permission to use as free online adaptation (resulting from e-mail answer)	Free usage in digital format (final descision)	Number of items	Informant	Age range	Clinical Uses
Overall mental health		MHC-SF	Mental Health Continuum-Short Form	Clinical use allowed	Yes	Contacting author not required	Possible	14	Y	12 - 18	S
Overall mental health		OHIO Scales for Youth	OHIO Scales for Youth	Clinical use allowed	Yes	Contacting author not required	Possible	48	Y, P, C	5 - 18	O
Overall mental health		PROMIS PGH-7	PROMIS pediatric global scale	Special conditions	Yes	Unclear	Unclear	7-9	Y, P	5 - 17	S, 0
Overall mental health		PSC	Pediatric Symptom Checklist	Clinical use allowed	No sufficient information	Permission granted	Possible	17-35	Y, P	3 - 17	S, 0
Overall mental health		SDQ	Strength and Difficulties Questionnaire	Special conditions	No sufficient information	Permission not granted	Not possible	25	Y, P, T	2 - 17	S, O
Overall mental health		SFSS	Symptoms and Functioning Severity Scale	No sufficient information	No sufficient information	Failed contacting	Not possible	26	Y, P, C	11 - 18	O
Overall mental health		TPA	Top Problems Assessment – Youth Report Form	Clinical use allowed	Yes	Contacting author not required	Possible	6	Y, P	5 - 15	D, O
Overall mental health		YP-CORE	Young Person's Clinical Outcomes in Routine Evaluation	Clinical use allowed	Yes	Contacting author not required	Possible	10	Y	11 - 16	S
Personality Disorder	BPD	Ab- DIB	Abbreviated version of the diagnostic interview for borderlines revised	No sufficient information	No sufficient information	Unclear	Unclear	26	Y	16 - 19	D
Personality Disorder	BPD	BPFS-C	Borderline Personality Features Scale for Children	No sufficient information	No sufficient information	Permission granted	Possible	24	Y	9 - 18	D
Personality Disorder	BPD	BPFS-C-11	Borderline Personality Features Scale for Children - 11	No sufficient information	No sufficient information	Permission granted	Possible	11	Y	9 - 18	D
Personality Disorder	BPD	BPFS-P	Borderline Personality Features Scale for Children – Parent Report	No sufficient information	No sufficient information	Permission granted	Possible	24	P	9 - 18	D
Personality Disorder	BPD	BSL-23	Borderline Symptom List - 23	No sufficient information	No sufficient information	Permission granted	Possible	23	Y	15 - 25	D
Personality Disorder	Criterion A	LPFS BF 2.0	Levels of Personality Functioning Scale Brief Form	No sufficient information	No sufficient information	Permission granted	Possible	12	Y	12 - 17	S
Personality Disorder	BPD	MBPD	Minnesota Borderline Personality Disorder Scale	No sufficient information	No sufficient information	Permission not granted	Not possible	19	Y	14 - 24	D

Table A.1. List of all instruments with their classification regarding free usage and adaptability to digital format.
This table presents the final list of instruments built as an update on a recent review by Becker-Haimes et al.[17], who reported on free, brief, and accessible instruments for assessing mental health in adolescents. Y: Youth; P: Parent: C: Clinician; T: Teacher; D: Diagnosis; O: Outcome monitoring; S: Screening.

Table A.1. continued

Domain	Personality disorder sub-domain	Abbreviation	Measure Name	Free (copyright license info available on the internet)	Software adaptation allowed (copyright license info available on the internet)	Copyright holder permission to use as free online adaptation (resulting from e-mail answer)	Free usage in digital format (final descision)	Number of items	Informant	Age range	Clinical Uses
Personality Disorder	BPD	MSI-BPD	McLean Screening Instrument for Borderline Personality Disorder – Self Report	No sufficient information	No sufficient information	Permission not granted	Not possible	10	Y	12 - 17	S
Personality Disorder	BPD	PAI-A-BOR	Personality Assessment Inventory–Borderline subscale	Not free	No sufficient information	Contacting author not required	Not possible	24	Y	12 - 18	D
Personality Disorder	BPD	PDQ+-4 BPD subscale	Personality Diagnostic Questionnaire-4+ (BPD subscale)	Not free	No sufficient information	Contacting author not required	Not possible	9	Y	14 - 18	D
Personality Disorder	Criterion B	PID-5-BF	The Personality Inventory for DSM-5—Brief Form (PID-5-BF)— Child Age 11–17	Clinical use allowed	No sufficient information	Permission granted	Possible	25	Y	11 - 17	S
Personality Disorder	Criterion B	PID-5+MA	Personality Inventory for DSM-5 and ICD-11 -Adolescent version	Clinical use allowed	No sufficient information	Permission granted	Possible	38	Y	12 - 18	S
Personality Disorder	Criterion A	SASPD	Standardized Assessment of Severity of Personality Disorder	No sufficient information	No sufficient information	Permission granted	Possible	9	Y	16 - 92	S
Psychosis		BCFRS	Bush-Francis Catatonia Rating Scale	No sufficient information	No sufficient information	Failed contacting	Not possible	23, 14	C	11 - 18	S
Psychosis		CAPE	Community Assessment of Psychic Experiences	Clinical use allowed	No sufficient information	Permission granted	Possible	42	Y	11 - 18	S
Psychosis		PQ-B	Prodromal Questionnaire Brief	No sufficient information	No sufficient information	Permission granted	Possible	21	Y	12 - 12	S
Substance Use		AID	CAGE Adapted to Include Drugs	Clinical use Allowed	Yes	Contacting author not required	Possible	4	Y,P	12 - 18	S
Substance Use		ANSS	Adolescents' Need for Smoking Scale	No sufficient information	No sufficient information	Failed contacting	Not possible	35	Y	11 - 18	S, D
Substance Use		BSTAD	Brief Screener for Tobacco Alcohol and Other Drugs	No sufficient information	No sufficient information	Failed contacting	Not possible	11	Y	12 - 17	S

Table A.1. List of all instruments with their classification regarding free usage and adaptability to digital format.
This table presents the final list of instruments built as an update on a recent review by Becker-Haimes et al.[17], who reported on free, brief, and accessible instruments for assessing mental health in adolescents. Y: Youth; P: Parent: C: Clinician; T: Teacher; D: Diagnosis; O: Outcome monitoring; S: Screening.

Table A.1. continued

Domain	Personality disorder sub-domain	Abbreviation	Measure Name	Free (copyright license info available on the internet)	Software adaptation allowed (copyright license info available on the internet)	Copyright holder permission to use as free online adaptation (resulting from e-mail answer)	Free usage in digital format (final descision)	Number of items	Informant	Age range			Clinical Uses
Substance Use		CPQ-A	Adolescent Cannabis Problems Questionnaire	No sufficient information	No sufficient information	Permission granted	Possible	12, 27	Y	14	-	18	S, D
Substance Use		CRAFFT	Car-Relax-Alone-Forget-Family-and-Friends-Trouble	No sufficient information	No sufficient information	Unclear	Unclear	9	Y, C	14	-	18	S
Substance Use		CUDIT-R	Cannabis Use Disorder Identification Test-Revised	Clinical use allowed	Yes	Contacting author not required	Possible	8	Y	11	-	18	S, D, O
Substance Use		CUPIT	Cannabis Use Problems Identification Test	No sufficient information	No sufficient information	Permission granted	Possible	16	Y	13	-	13	S
Substance Use		FTND	Fagerstrom Test for Nicotine Dependence	Clinical use allowed	Yes	Contacting author not required	Possible	7	Y	11	-	18	S
Substance Use		HONC	Hooked on Nicotine Checklist	Clinical use allowed	No sufficient information	Failed contacting	Unclear	10	Y	12	-	12	S, D, O
Substance Use		RAPI	Rutgers Alcohol Problem Index	Clinical use allowed	Yes	Contacting author not required	Possible	10, 18, 23	Y, C	12	-	18	S, D
Substance Use		RCQ	Risks and Consequences Questionnaire	Non-commercial use	Yes	Permission granted	Possible	30	Y	14	-	19	D
Substance Use		SDS	Severity Dependence Scale	No sufficient information	No sufficient information	Permission granted	Possible	5	Y	14	-	14	S
Substance Use		TCU Drug Screen	TCU Drug Screen	Non-commercial use	No sufficient information	Failed contacting	Not possible	15	Y	13	-	19	S
Suicidality		ABUSI	Alexian Brothers Urge to Self-Injure Scale	Clinical use allowed	No sufficient information	Permission granted	Possible	5	Y	11	-	18	D, O
Suicidality		ASQ	Ask Suicide-Screening Questions	Clinical use allowed	No sufficient information	Failed contacting	Unclear	4	C	10	-	24	S
Suicidality		C-SSRS	Columbia Suicide Screen Severity Rating Scale	No sufficient information	No sufficient information	Permission granted	Possible	19	C	5	-	18	S, D
Suicidality		DSI-SS	Depressive Symptom Inventory Suicidality Subscale	No sufficient information	No sufficient information	Failed contacting	Not possible	4	Y	15	-	15	S, O
Suicidality		FASM	Functional Assessment of Self-Mutilation	No sufficient information	No sufficient information	Failed contacting	Not possible	40	Y	11	-	18	D

Table A.1. List of all instruments with their classification regarding free usage and adaptability to digital format.
This table presents the final list of instruments built as an update on a recent review by Becker-Haimes et al.[17], who reported on free, brief, and accessible instruments for assessing mental health in adolescents. Y: Youth; P: Parent: C: Clinician; T: Teacher; D: Diagnosis; O: Outcome monitoring; S: Screening.

Table A.1. continued

Domain	Personality disorder sub-domain	Abbreviation	Measure Name	Free (copyright license info available on the internet)	Software adaptation allowed (copyright license info available on the internet)	Copyright holder permission to use as free online adaptation (resulting from e-mail answer)	Free usage in digital format (final decision)	Number of items	Informant	Age range	Clinical Uses
Suicidality		SBQ-R	Suicidal Behaviors Questionnaire Revised	No sufficient information	No sufficient information	Failed contacting	Not possible	4	C	15 - 18	S
Traumatic Stress		CATS	Child and Adolescent Trauma Screen	Clinical use allowed	No sufficient information	Permission granted	Possible	20	Y,P	3 - 17	S
Traumatic Stress		CPSS-5-SR	Child PTSD Symptom Scale	Non-commercial use	Yes	Failed contacting	Not possible	6,24	Y	8 - 18	S,D
Traumatic Stress		CPTCI	Child Post-traumatic Cognitions Inventory	No sufficient information	No sufficient information	Permission granted	Possible	25	Y	6 - 18	S, D
Traumatic Stress		CRIES	Children’s Revised Impact of Event Scale	Clinical use allowed	No sufficient information	Permission granted	Possible	8, 13	Y	8 - 18	S, D
Traumatic Stress		CSDC	Child Stress Disorders Checklist	Non-commercial use	No sufficient information	Failed contacting	Not possible	36	P	2 - 18	S, D
Traumatic Stress		CTS	Child Trauma Screen (CTS) by Jason M. Lang	No sufficient information	No sufficient information	Permission granted	Possible	10	Y, P	6 - 17	S
Traumatic Stress		CTSQ	Child Trauma Screen, check Becker-Haimes, this is the original entry form out list	Special conditions	No sufficient information	Unclear	Unclear	10	Y	7 - 16	S, D
Traumatic Stress		CYRM	Child and Youth Resilience Measure Revised	Special conditions	No sufficient information	Permission granted	Possible	17	Y, P	5 - 23	D

Table A.1. List of all instruments with their classification regarding free usage and adaptability to digital format.
This table presents the final list of instruments built as an update on a recent review by Becker-Haimes et al.[17], who reported on free, brief, and accessible instruments for assessing mental health in adolescents. Y: Youth; P: Parent: C: Clinician; T: Teacher; D: Diagnosis; O: Outcome monitoring; S: Screening.
