# Supplementary material for: Digital use of standardised assessment tools for children and adolescents: can available paper-based questionnaires be used free of charge in electronic format?
Source: BMC Psychiatry. 2022 Jun 3;22:379. doi: 10.1186/s12888-022-04023-w (PMC9166519; doi:10.1186/s12888-022-04023-w)
Supplement: Supplementary file 2 — Additional file 2. [file 12888_2022_4023_MOESM2_ESM.pdf]

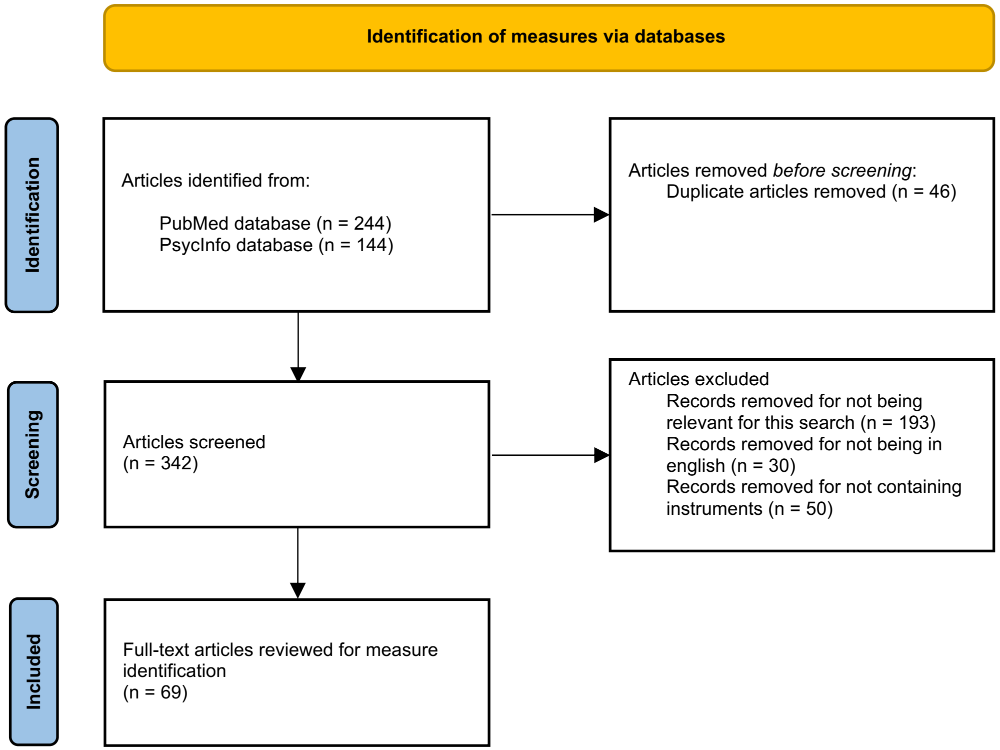

Figure A.1: PRISMA Flow Diagram for systematic literature search on child and adolescent measures for personality disorder.
