# Supplementary material for: Digital use of standardised assessment tools for children and adolescents: can available paper-based questionnaires be used free of charge in electronic format?
Source: BMC Psychiatry. 2022 Jun 3;22:379. doi: 10.1186/s12888-022-04023-w (PMC9166519; doi:10.1186/s12888-022-04023-w)
Supplement: Supplementary file 3 — Additional file 3. [file 12888_2022_4023_MOESM3_ESM.pdf]

Dear Sir or Madam /Ms./Mr.,

We are an international research group working on a project that is promoting the use of evidence-based assessments especially in Low- and Middle-Income Countries (LMIC). We believe that an electronic implementation is required especially in lower resource contexts.
The project is currently conducted in a collaboration of University of Basel (Switzerland), Muhimbili University of Health and Allied Sciences (Tanzania), University of Pristina (Kosovo) and the Millennium Institute for Research in Depression and Personality (Chile).

Our project aims at creating and providing an open source platform 'Mental Health Information Reporting Assistant' (MHIRA, https://mhira-project.org/). The platform is planned to be made available to hospitals, private practices as well as for school psychologists.

We are currently preparing a review on freely available health care measures for adolescent psychiatry. Such a review has recently been published (Becker-Haimes et al., 2020) and cites the instrument(s) XXX. Beyond this review, we are interested in the usage of the measures in an open source platform in a digital format.

Our approach is to deliver MHIRA without content i.e. without integrated questionnaires or instruments but with the idea that clinicians and hospitals select the tools that they would like to use. For this purpose, the platform contains a questionnaire builder which would allow clinicians to integrate available instruments to be used at a specific mental health care center. The data provided by these measures would be used to generate automatic reports for clinical use.

The instruments and reports could be integrated while maintaining the content and the required copyright statement. However the instruments could look different as we cannot guarantee a replication of the format for each questionnaire.
For most of the contexts we consider, a translation and cultural adaptation of the tools would be required and it is our aim to support centers in the execution of this task.

MHIRA has a dedicated mandatory field for copyright information and references to your instrument can be added. Thus, we provide the necessary means for proper credit of your work in the software.

For our review and, later, a potential clinical implementation, we would like to know whether the use of your instrument would be allowed and what the conditions would be. Please let us know whether an electronic format of your instrument is already available.

Thank you very much in advance for considering our project and we are looking forward to hearing your feedback. Please do not hesitate to contact us in case of any questions.

[redacted]

**Figure A.2** Email template used for contacting the authors for permission regarding free usage for clinical purposes and allowed software adaptation.
